# Shikonin inhibits the growth of human prostate cancer cells via modulation of the androgen receptor

**DOI:** 10.3892/ijo.2014.2306

**Published:** 2014-02-20

**Authors:** SOON YOUNG JANG, EUN HYANG JANG, SEO YOUNG JEONG, JONG-HO KIM

**Affiliations:** 1 Departments of Pharmaceutical Science, College of Pharmacy, Kyung Hee University, Dongdaemun-gu, Seoul 130-701, Republic of Korea; 2 Life and Nanopharmaceutical Science, Kyung Hee University, Dongdaemun-gu, Seoul 130-701, Republic of Korea

**Keywords:** Shikonin, androgen receptor, prostate cancer, anticancer drug

## Abstract

Shikonin, a natural naphthoquinone isolated from the traditional Chinese medicine *Zi Cao (gromwell)*, has been shown to possess tumor cell killing activity. The human androgen receptor (AR) is a nuclear transcription factor that serves as a major therapeutic target for prostate cancer. However, AR regulation by shikonin has not been reported. We investigated the effects of shikonin on the growth of prostate cancer cells. We observed that shikonin decreased the expression of AR at both the mRNA and the protein levels in LNCaP and 22RV1 human prostate cancer cells. The results from a luciferase assay showed that shikonin decreased the transcriptional activity of AR. Moreover, shikonin treatment inhibited AR target gene expression, PSA and growth inhibition of prostate cancer cells. In conclusion, the present study shows for the first time that shikonin treatment causes transcriptional repression of AR and inhibition of its nuclear localization in human prostate cancer cells. We propose that shikonin, an anticancer drug extracted from natural sources, induces inhibition of cell growth through modulation of AR in androgen-responsive prostate cancer cells and is a candidate for use in cancer chemotherapy for human prostate cancer.

## Introduction

Prostate cancer is the most frequently diagnosed non-cutaneous male malignancy and the third leading cause of cancer-related death in men in most western industrialized countries (1). Since it is estimated that around 660,000 men worldwide will be diagnosed with prostate cancer it will remain a major health problem in coming years (1). Despite an initial efficacy of androgen deprivation therapy, most patients with prostate cancer progress within 2 years from androgen-dependent status to hormone-refractory prostate cancer, for which there is no curative therapy. Androgen receptor (AR) signaling plays a key role in the development of hormone-refractory prostate cancer. AR, a member of the steroid receptor superfamily, is a ligand-dependent transcription factor that mediates androgen action in cells. AR is composed of three major domains: an NH2-terminal transcriptional activation domain (NTD), a central DNA-binding domain, and a COOH-terminal ligand-binding domain (LBD) (2,3). AR is associated with cellular chaperones in the cytosol in its inactive state (4). After binding to androgens, such as testosterone and, more potently, dihydrotestosterone (DHT), AR translocates to the nucleus, binds to the androgen response elements of AR target gene promoter, and regulates expression of AR target genes (2,5). AR hypersensitivity, as a result of AR gene mutation and/or amplification, overexpression of coactivators, and AR cross-talking with other signal transduction pathways, often occurs and plays crucial roles in prostate cancer development, progression, and androgen-independent growth. In other words, AR and its signaling axis are the most important targets for therapies against advanced prostate cancer (5–7). Therefore, finding novel and more effective inhibitors of AR signaling is of great interest.

Shikonin is an active naphthoquinone compound and the main component of red pigment extracts from the Chinese medicinal herb, *Lithospermum erythrorhizon* Sieb et Zucc. Shikonin and its analogues can kill cancer cells through a number of mechanisms, including inhibition of topoisom-erase-I (8), polo-like kinase 1 (PLK1) and protein tyrosine kinase (PTK) (9); regulation of phosphorylation-dependent activities of extracellular-regulated protein kinase (pERK), c-Jun N-terminal kinase (JNK), and protein kinase Cα PKCα (10); suppression of tumor necrosis factor receptor-associated protein 1 (TRAP1) expression (11); activation of caspases (12); and inhibition of proteasome activity (13). In previous studies, shikonin and its derivatives were shown to exert anti-proliferative and pro-apoptotic effects against some tumor cells, including sarcoma 180 (S-180) ascites cells, gastric cancer, colon adenocarcinoma, and oral cancer (14). A recent report has also shown that shikonin activates p53 and caspase-9 pathways (15) in human malignant melanoma A375-S2 cells.

In this study, we hypothesized that shikonin may have a role as an inhibitor of AR signaling and, thus, could serve as a therapeutic agent for the management of human prostate cancers. We report strong anti-AR activity of the natural product shikonin in prostate cancer cells. Shikonin markedly decreased expression not only of AR, but also PSA, a widely used serologic marker for prostate cancer burdens and an indicator of therapeutic efficacy and recurrence (16), and inhibited growth of AR-positive human prostate cancer cells. We propose that shikonin is a good candidate for use as an anticancer drug in chemoprevention and treatment of hormonal-responsive prostate tumor cells.

## Materials and methods

### Reagents

RPMI-1640 was purchased from Gibco Technologies, Inc. (Gaithersburg, MD, USA). Fetal bovine serum (FBS) was obtained from Hyclone (Logan, UT, USA). Shikonin was purchased from Sigma-Aldrich (St. Louis, MO, USA). Antibodies for AR, β-actin, PSA, PCNA, Bcl-2, PARP, and anti-goat peroxidase conjugated secondary antibody were obtained from Santa Cruz Biotechnology (Santa Cruz, CA, USA). Anti-mouse and anti-rabbit peroxidase conjugated secondary antibodies were purchased from Pierce (Madison, WI, USA). The dual-luciferase reporter assay kit was from Promega (Madison, WI, USA).

### Cell culture

LNCaP and 22RV1 prostate cancer cells were maintained in RPMI-1640 supplemented with 10% FBS and 1% penicillin/streptomycin antibiotics.

### Immunoblotting

Following reagent treatments, cells were washed twice with 1X PBS and cell extracts were prepared using RIPA buffer (1X PBS, 1% NP40, 0.5% sodium deoxycholate, 0.1% SDS containing an additional 100 *μ*l of 10 mg/ml PMSF, and 1 tablet of the complete mini protease inhibitors). Protein content was quantified by the Lowry assay. Lysate proteins were resolved by SDS-PAGE and transferred onto a nitrocellulose membrane. The membrane was incubated with TBS buffer containing 0.1% Tween-20 and 5% skim milk, and then exposed to the desired primary antibody. After treatment with a proper secondary antibody, the immunoreactive bands were visualized by standard ECL method.

### Cell fractionation

Nuclear and cytoplasmic fractions were prepared using nuclear and cytoplasmic extraction reagents kit (Fermentas, St. Leon-Rot, Germany). Cell lysates were spun down for 7 min at 500 × g, and the supernatant was designated the cytoplasmic fraction. The nuclear pellet was washed three times in wash buffer and then treated with nuclear lysis buffer for 15 min. The sample was spun at 20,000 × g for 15 min, and the supernatant designated the nuclear fraction. PCNA and β-actin were used as markers for nuclear and cytoplasmic proteins.

### Real-time PCR and quantitative real-time PCR (qRT-PCR)

Total RNA isolation was performed with the use of the TRIzol (Invitrogen) according to the manufacturer’s protocol. The cDNA was synthesized with the use of 2 mg of total RNA through SuperScript reverse transcriptase (Bioneer, Daejeon, Korea) with oligo dT primers. PCR was done with a specific primer (AR sense 5′-ATGGTGAGCAGAGTGCCCTA-3′; antisense 5′-GTGGTGCTGGAAGCCTCTCCT-3′; GAPDH sense, 5′-GGCCTCCAAGGAGGAAGACC-3′; and GAPDH antisense, 5′-AGGGGTCTACATGGCAACTG-3′). GAPDH, a non-regulated housekeeping gene was used as an internal control to normalize input cDNA. RT-PCR was performed using the LightCycler 480 using SYBR green master mix (Roche). Each experiment was performed in three experimental replicates with three technical replicates within each experiment.

### Luciferase reporter assay

LNCaP and 22RV1 cells were transiently co-transfected with 0.5 *μ*g ARE-, PSA-luciferase plasmid and 0.5 mg pSV-β-galactosidase reporter vector using Lipofectamine 2000 (Invitrogen) transfection reagent. After transfection for 24 h, cells were treated with shikonin for the described period. Cell extracts were prepared for the luciferase assays. The luciferase activity was normalized by β-galactosidase activity.

### Cell proliferation and viability assays

All proliferation assays were based on the MTT method. Cells were seeded in a 96-well plate, 1×10^4^ cells per well. After 24–72 h, cells were treated with shikonin. At the end of the experiment, the media was removed and DMSO was added as MTT solubilization solution. Absorbance was measured at 550 nm.

## Results

### Shikonin decreases the AR protein level in LNCaP and 22RV1 cells

**Figure 1.**
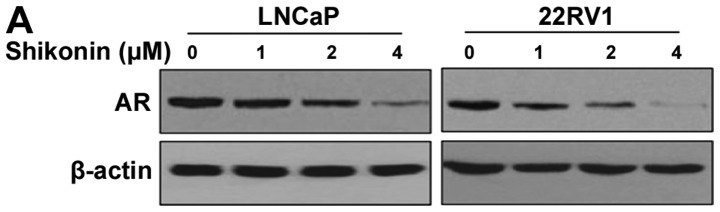

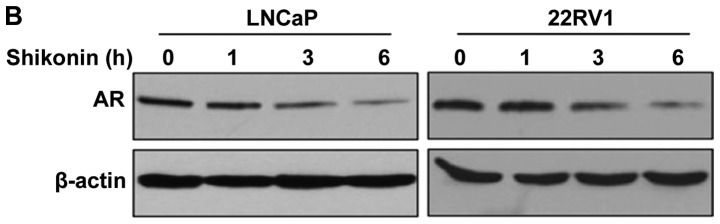
Shikonin decreases the AR protein level in LNCaP and 22RV1 cells. (A and B) LNCaP and 22RV1 cells were treated with various concentrations of shikonin for 6 h (A) and 4 *μ*M shikonin for the indicated time-points (B). Cell lysates were separated on 10% SDS-polyacrylamide gels (30 *μ*g/lane) and the AR protein level was detected by western blotting with anti-AR antibody. The membranes were stripped and reprobed with anti-β-actin antibody. β-actin was used as an internal control.

**Figure 2.**
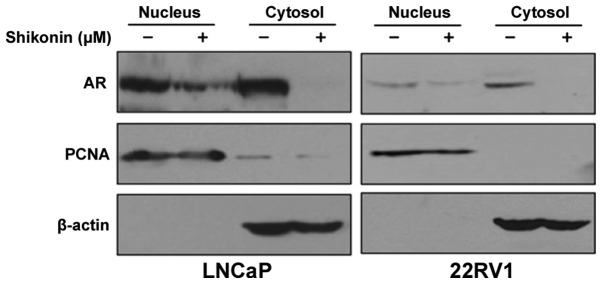
Shikonin decreses AR protein expression in both cytoplasmic and nuclear fractions. LNCaP cells were treated with 4 *μ*M shikonin for 6 h. Cells were harvested and fractionated into the cytoplasm and the nucleus. Samples were then separated on 10% SDS-polyacrylamide gels and subjected to western blotting with anti-AR antibody. The analysis was repeated three times, and β-actin and PCNA were used as markers for the cytoplasmic and nuclear fractions.

In androgen-responsive prostate cancer cells, AR is required for the initiation of androgen-dependent gene transcription. We examined the effect of shikonin on the expression of AR in the androgen-responsive human prostate cancer cell lines LNCaP and 22RV1. Porstate cancer cells were treated with shikonin at various concentrations and subjected to western blotting. Shikonin decreased the protein level of AR in a dose-dependent manner in both cell lines (Fig. 1A). The AR protein level started to decrease with 2 *μ*M shikonin and almost disappeared with 4 *μ*M shikonin (Fig. 1A). We also examined the time-dependent effect of shikonin on AR. The AR protein level decreased in a time-dependent manner in prostate cancer cells treated with 4 *μ*M shikonin (Fig. 1B). Next, we examined whether shikonin affects translocation of AR to the nucleus, because nuclear localization of AR is important for its transcriptional activity (17). LNCaP and 22RV1 cells were treated with 4 *μ*M shikonin and fractionated into cytoplasmic and nuclear fractions. Shikonin decreased the AR protein levels in both the nucleus and cytoplasm (Fig. 2). These results indicated that shikonin decreases the AR protein level in a dose-, time-dependent manner and also, effectively block nuclear localization of AR in LNCaP and 22RV1 prostate cancer cells.

### Shikonin suppresses the mRNA level of AR in LNCaP and 22RV1 cells

**Figure 3.**
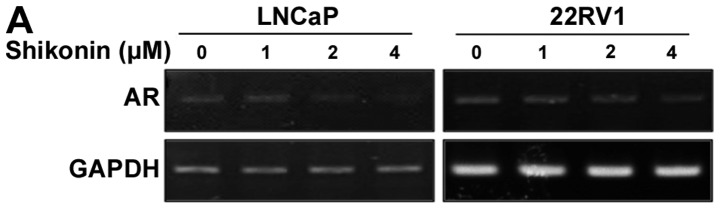

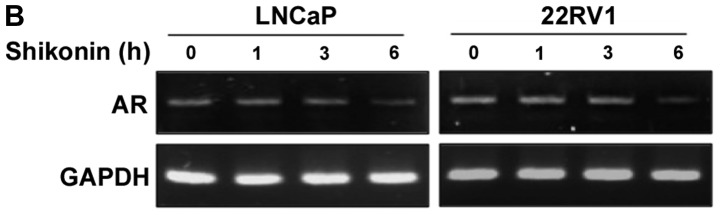

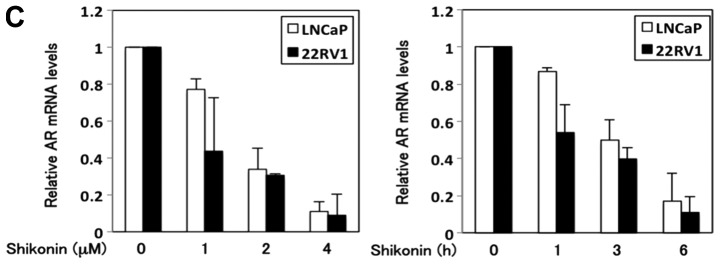
Shikonin suppresses the mRNA level of AR in LNCaP and 22RV1 cells. (A and B) LNCaP and 22RV1 cells were treated with various concentrations of shikonin for 6 h (A) and 4 *μ*M shikonin for the indicated time-points (B). Total RNA was extracted from cells and RT-PCR analysis was performed using primers specific for AR. The PCR products were separated on a 1% agarose gel. GAPDH was used as an internal control. (C) LNCaP and 22RV1 cells were treated with different concentrations of shikonin for 6 h and 4 *μ*M Shikonin for the indicated time-points. RNA isolation and real-time PCR were performed as described in Materials and methods. Results are expressed as the mean ± SEM of three replicate measurements from a single experiment and it is representative of three separate experiments.

Since shikonin decreased the AR protein expression, we examined the effect of shikonin to mRNA level of AR in LNCaP and 22RV1 cells. The mRNA level of AR was determined using semi-quantitative RT-PCR. Cells treated with shikonin at various concentrations for 6 h exhibited a marked and concentration-dependent decrease in AR mRNA levels (Fig. 3A). The mRNA level of AR in cells treated with 4 *μ*M shikonin for 6 h was decreased to 10% of the level compared to without shikonin treatment (Fig. 3A). Shikonin also decreased the AR mRNA level in a time-dependent manner in LNCaP and 22RV1 cells (Fig. 3B). As shown in Fig. 3C, shikonin decreased the AR mRNA expression dose-and time-dependently. These results indicate that shikonin decreases the expression of AR at the transcriptional level.

### Shikonin decreased the transcriptional activity of AR

**Figure 4.**
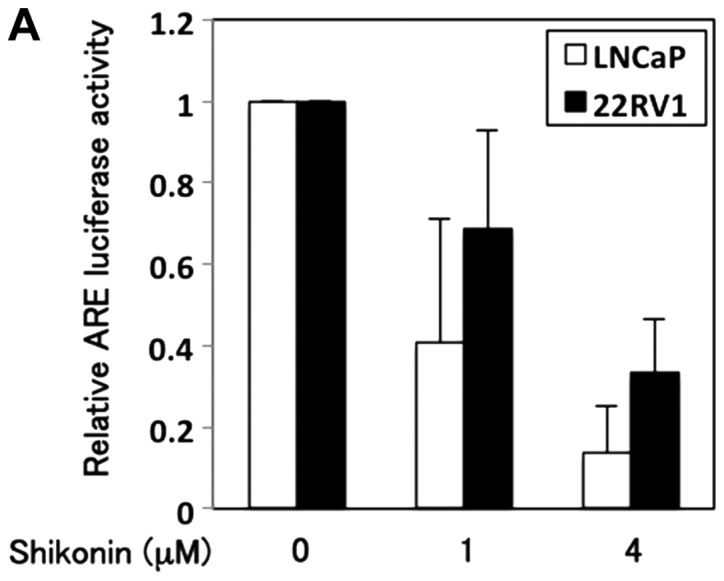

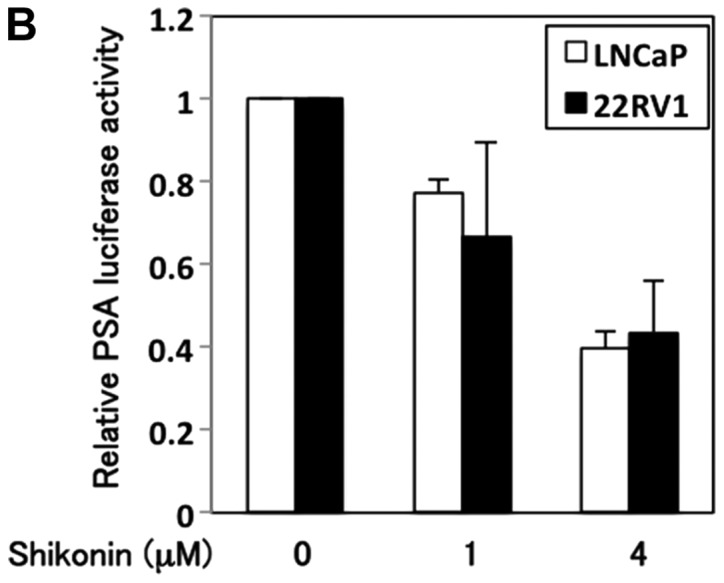
Shikonin decreases the transcriptional activity of AR. (A and B) LNCaP and 22RV1 cells were transfected with the ARE-, PSA-luciferase reporter gene plasmid (0.5 *μ*g). After 24 h, cells were treated with the indicated concentrations of shikonin for 6 h and harvested for the luciferase assays. The luciferase activity was normalized by β-galactosidase activity, and the experiments were performed in triplicate. Data are expressed as the mean ± SD and are presented as the relative luciferase activity.

AR is an important transcription factor that regulates expression of a variety of target genes that harbor the androgen response element (ARE) in their promoters (17,18). Therefore, we examined the effect of shikonin on the transcriptional activity of AR. LNCaP and 22RV1 cells were transfected with an ARE-containing promoter reporter gene. AR transactivation was determined using a luciferase assay in cells treated with or without shikonin. Shikonin decreased the luciferase activity in a dose-dependent manner (Fig. 4A). Cells treated with 4 *μ*M shikonin showed ∼5-fold and 2.5-fold decrease in the AR transcriptional activity in LNCaP and 22RV1 cells, respectively. To further determine the inhibitory effects of shikonin on PSA gene transcription, the PSA promoter-driven luciferase reporter activity, was assessed in prostate cancer cells transfected with pGL3-PSA-luc. PSA promoter activity was reduced when the cells were treated with shikonin (Fig. 4B). These results indicated that the shikonin mediated decrease in AR protein level correlated with repression of its promoter activity.

### Inhibition of AR transcriptional activity by shikonin leads to suppression of AR-induced gene expression

**Figure 5.**
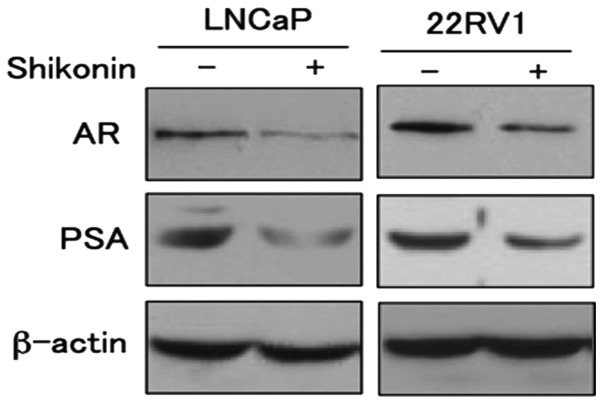
Inhibition of AR transcriptional activity by shikonin leads to suppression of AR-induced gene expression. LNCaP and 22RV1 cells were treated with or without 4 *μ*M shikonin for 6 h. The protein levels were detected by western blotting with anti-AR antibody and anti-PSA antibody. The membranes were stripped and reprobed with anti-β-actin antibody. β-actin was used as an internal control.

To determine the effect of decreased AR transcriptional activity by shikonin, we examined the expressions of the AR target gene PSA (Fig. 5). LNCaP and 22RV1 cells were treated with shikonin and subjected to western blotting. The protein expression levels of PSA decreased in response to shikonin in a dose-dependent manner (data not shown). The expression of prostate specific antigen (PSA) has been used extensively as a marker of prostate cancer growth and is a well-known target gene of AR. These results indicate that shikonin decrease the transcriptional activity of AR leading to suppression of the target protein PSA expression.

### Shikonin inhibits the growth of LNCaP and 22RV1 cells

**Figure 6.**
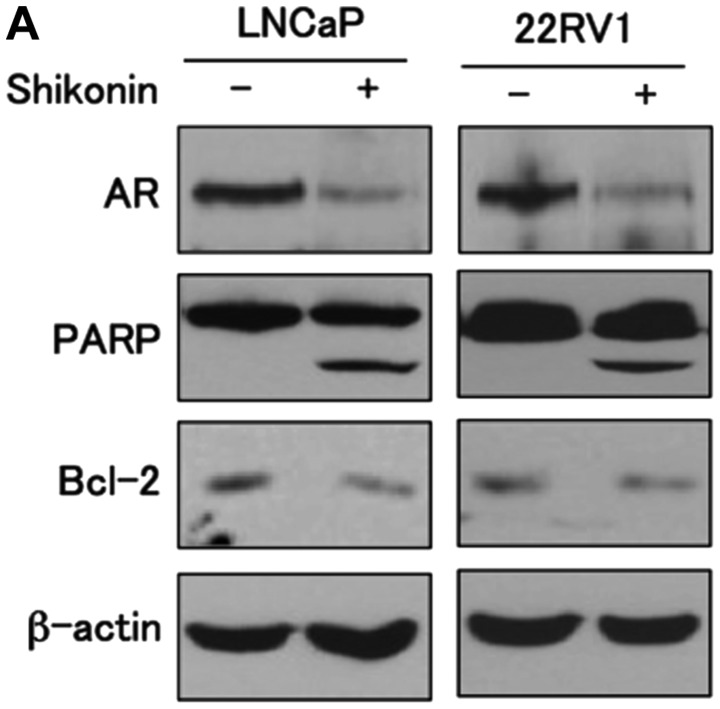

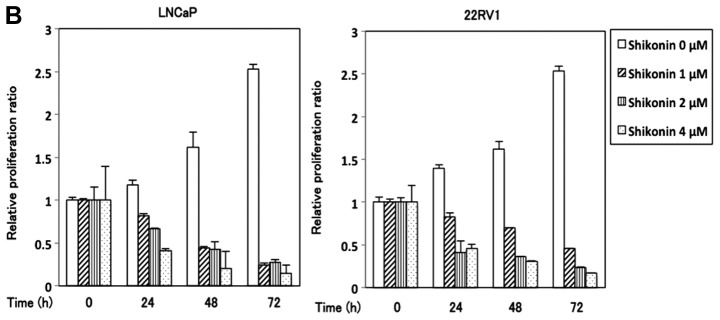
Shikonin inhibits AR-positive prostate cancer cell growth. (A) LNCaP and 22RV1 cells were treated with or without 4 *μ*M shikonin for 6 h. The protein levels were detected by western blotting with anti-PARP and anti-Bcl-2 antibodies. The membranes were stripped and reprobed with anti-β-actin antibody. β-actin was used as an internal control. (B) LNCaP and 22RV1 cells were seeded in 96-well plates and treated with various dose of shikonin for the indicated time-points. The cell proliferation was determined by the MTT assay. Data were presented as the percentage of proliferation relative to medium-treated control. All measurements were performed in triplicate.

AR is an important regulator of cellular growth in androgen-dependent prostate cancer cells. Shikonin suppresses AR expression at the transcriptional level and subsequently inhibits the transcriptional activity of AR, resulting in inhibition of cell proliferation in androgen-positive prostate cancer cells. Therefore, we next examined whether shikonin affects the growth of prostate cancer cells. LNCaP and 22RV1 cells were treated with or without 4 *μ*M shikonin for 6 h and the levels of proteins involved in apoptosis were examined. Cells treated with shikonin increased cleavage of poly(ADP-ribose) polymerase (PARP) cleavage and decreased expression of the anti-apoptotic molecule Bcl-2 compared to cells without shikonin in both cell lines (Fig. 6A). We next examined the effect of shikonin on the growth of LNCaP and 22RV1 cells. Cell proliferation assays were determined using MTT analysis. As shown in Fig. 6B, the viability of prostate cancer cells treated with 4 *μ*M shikonin for 24 h was reduced in a time-dependent manner to ∼50% of the viability of control cells. These data are consistent with the induction of apoptosis by the shikonin as indicated by the PARP cleavage in both LNCaP and 22RV1 cells treated with shikonin. Taken together, these results indicate that shikonin led to growth inhibition of AR-positive prostate cancer cells thereby suppressing the transcriptional and translational level of AR.

## Discussion

Prostate cancer is a leading cause of cancer-related deaths among men in the United States (19). The mechanism underlying the pathogenesis of prostate cancer is not fully understood, but age, race, dietary habits, and androgen secretion and metabolism are some of the risk factors associated with this malignancy (20). AR, a ligand-activated transcription factor belonging to the steroid receptor super-family, is critically involved in prostate cancer progression as well as maintenance of the male reproductive organ (17). Ligand-free AR predominantly resides in the cytoplasm complexed with chaperone proteins, including Hsp90, but in a conformational state responsive to ligand binding (17). Ligand-activated regulation of AR leads to its subsequence events such as nuclear translocation, dimerization, and binding to androgen response elements in the DNA of target genes (21). Moreover, AR is assumed to be a major molecule in the transition from hormone-sensitive to androgen-independent prostate cancer (22). It is hormone ablation therapy that is the main treatment for early-stage prostate cancer. However, this treatment leads to incurable and acute hormone refractory disease. Therefore, it is necessary for novel strategies to effectively eliminate AR signaling from prostate cancer for the clinical control of this lethal disease. Herein, the shikonin-mediated down-modulation in AR protein level correlates with a reduction in AR message as presented by reverse transcription-PCR and inhibition of AR promoter activity as revealed by the luciferase reporter assay, which is supported by the following observations: i) nuclear level of AR is markedly suppressed in the presence of shikonin in both LNCaP and 22RV1 cell lines; ii) shikonin treatment results in a critical decrease in expression levels of the AR-regulated target gene PSA; and iii) shikonin inhibits growth of LNCaP and 22RV1 cells in association with apoptosis induction.

Shikonin is the main component of Chinese herbal medicine *Zi Cao (gromwell)* that has antitumor activity (23). Although several *in vitro* molecular targets were found to be associated with shikonin-induced apoptotic cell death (14,24), the cellular target of shikonin is still unknown. Natural compounds might have multiple cellular targets in order to achieve their biological beneficial effects such as tumor growth inhibition (25).

In this study, we firstly describe that the AR is one of the targets of shikonin *in vitro*, inhibition of which leads to cell death in human prostate tumor cells, shikonin is highly effective in reducing the protein level of AR and the transcriptional activity of AR. Consequently, we suggest that shikonin has great potential to be used clinically for treatment of human prostate cancers.
